# Accuracy in Wrist-Worn, Sensor-Based Measurements of Heart Rate and Energy Expenditure in a Diverse Cohort

**DOI:** 10.3390/jpm7020003

**Published:** 2017-05-24

**Authors:** Anna Shcherbina, C. Mikael Mattsson, Daryl Waggott, Heidi Salisbury, Jeffrey W. Christle, Trevor Hastie, Matthew T. Wheeler, Euan A. Ashley

**Affiliations:** 1 Division of Cardiovascular Medicine, Department of Medicine, Stanford University, Stanford, CA 94305, USA; annashch@stanford.edu (A.S.); mikaelm@stanford.edu (C.M.M.); dwaggott@stanford.edu (D.W.); JChristle@stanfordhealthcare.org (J.W.C.); wheelerm@stanford.edu (M.T.W.); 2 Åstrand Laboratory of Work Physiology, The Swedish School of Sport and Health Sciences, Stockholm 114 33, Sweden; 3 Center for Inherited Cardiovascular Disease, Division of Cardiovascular Medicine, Stanford University, Stanford, CA 94305, USA; hsalisbury@stanfordhealthcare.org; 4 Department of Statistics, Stanford University, Stanford, CA 94305, USA; hastie@stanford.edu; 5 Department of Biomedical Data Science, Falk Cardiovascular Research Building, Stanford University, 870 Quarry Road, Stanford, CA 94305, USA

**Keywords:** mobile health, heart rate, energy expenditure, validation, fitness trackers, activity monitors

## Abstract

The ability to measure physical activity through wrist-worn devices provides an opportunity for cardiovascular medicine. However, the accuracy of commercial devices is largely unknown. The aim of this work is to assess the accuracy of seven commercially available wrist-worn devices in estimating heart rate (HR) and energy expenditure (EE) and to propose a wearable sensor evaluation framework. We evaluated the Apple Watch, Basis Peak, Fitbit Surge, Microsoft Band, Mio Alpha 2, PulseOn, and Samsung Gear S2. Participants wore devices while being simultaneously assessed with continuous telemetry and indirect calorimetry while sitting, walking, running, and cycling. Sixty volunteers (29 male, 31 female, age 38 ± 11 years) of diverse age, height, weight, skin tone, and fitness level were selected. Error in HR and EE was computed for each subject/device/activity combination. Devices reported the lowest error for cycling and the highest for walking. Device error was higher for males, greater body mass index, darker skin tone, and walking. Six of the devices achieved a median error for HR below 5% during cycling. No device achieved an error in EE below 20 percent. The Apple Watch achieved the lowest overall error in both HR and EE, while the Samsung Gear S2 reported the highest. In conclusion, most wrist-worn devices adequately measure HR in laboratory-based activities, but poorly estimate EE, suggesting caution in the use of EE measurements as part of health improvement programs. We propose reference standards for the validation of consumer health devices (http://precision.stanford.edu/).

## 1. Introduction

Coronary heart disease is responsible for one in every four deaths in the United States. Few interventions are as effective as physical activity in reducing the risk of death yet, we have achieved limited success in programs designed to help individuals exercise more. In weight loss studies, clear benefit derives from simple documentation of caloric intake, [1] but data are less clear on the benefit of documenting exercise time and calorie expenditure on health.

Microelectromechanical systems such as accelerometers and Light Emitting Diode (LED)-based physiological monitoring have been available for decades [2,3,4,5,6,7]. More recent improvements in battery longevity and miniaturization of the processing hardware to turn raw signals in real time into interpretable data led to the commercial development of wrist-worn devices for physiological monitoring. Such devices can provide data directly back to the owner and place estimates of heart rate (HR) and energy expenditure (EE) within a consumer model of health and fitness. Unlike clinically approved devices, however, validation studies are not available to practitioners whose patients commonly present acquired data in the hope that it may enhance their clinical care. Indeed, certain health care systems have developed processes to bring such data directly into the medical record [8,9,10]. Thus, validation data on new devices and a forum for the ready dissemination of such data are urgent requirements.

Prior studies of wrist-worn devices have focused on earlier stage devices, or have focused exclusively on HR or estimation of EE. Some have made comparisons among devices without reference to the U.S. a Food and Drug Administration (FDA) approved gold standard. None proposed an error model or framework for device validation. In response to this need, we formulated an approach to the public dissemination of validation data for consumer devices (http://precision.stanford.edu/). The website is one answer to the challenge of rapid technological advance and algorithm/product cycle upgrades. We present here the first data from this study, derived from laboratory testing of consumer wrist-worn devices from the most commercially successful manufacturers. We test devices in diverse conditions on diverse individuals, and present the data and recommendations for error modeling.

## 2. Methods

### 2.1. Devices

Following a comprehensive literature and online search, 45 manufacturers of wrist-worn devices were identified. Criteria for inclusion included: wrist-worn watch or band; continuous measurement of HR; stated battery life >24 h; commercially available direct to consumer at the time of the study; one device per manufacturer. Eight devices met the criteria; Apple Watch; Basis Peak; ePulse2; Fitbit Surge; Microsoft Band; MIO Alpha 2; PulseOn; and Samsung Gear S2. Multiple ePulse2 devices had technical problems during pre-testing and were therefore excluded. All devices were bought commercially and handled according to the manufacturer’s instructions. Data were extracted according to standard procedures described below.

Devices were tested in two phases. The first phase included the Apple Watch, Basis Peak, Fitbit Surge and Microsoft Band. The second phase included the MIO Alpha 2, PulseOn and Samsung Gear S2.

Healthy adult volunteers (age ≥18) were recruited for the study through advertisements within Stanford University and local amateur sports clubs. From these interested volunteers, study participants were selected to maximize demographic diversity as measured by age, height, weight, body mass index (BMI), wrist circumference, and fitness level. In total, 60 participants (29 men and 31 women) performed 80 tests (40 with each batch of devices, 20 men and 20 women). Participant characteristics are presented in Table 1.

Skin tone at the wrist was rated independently by two investigators using the Von Luschan Chromatic scale (1–36), and the average rating was then transformed to the Fitzpatrick skin tone scale (1–6) [11]. Maximal oxygen uptake (VO_2_max) was measured with the Quark CPET (COSMED, Rome, Italy) by incremental tests in running (*n* = 32) or cycling (*n* = 6) to volitional exhaustion, or estimated from the submaximal cycling stages (*n* = 22) using the Åstrand nomogram [12]. In the running test, the subject began the test running at 5.0 mph, 1% incline. Each minute, the speed was increased by 0.5 mph and the subject was asked to assess their exertion level using the Borg Rating of Perceived Exertion (RPE) scale [13]. In order to complete the test within a 10-minute period, the incline was increased by 0.5% each minute beginning at minute 7 until the subject reached volitional exhaustion. For subjects who performed the cycling test, initial resistance was set at 125 W and increased by 25 W each minute until volitional exhaustion. As with the running test, subjects rated their perceived exertion on the Borg RPE scale at the end of each minute.

The study was conducted in accordance with the principles outlined in the Declaration of Helsinki and approved by the Institutional Review Board of Stanford University (protocol ID 34651, Euan Ashley). All participants provided informed consent prior to the initiation of the study.

### 2.2. Protocol

**Figure 1:**
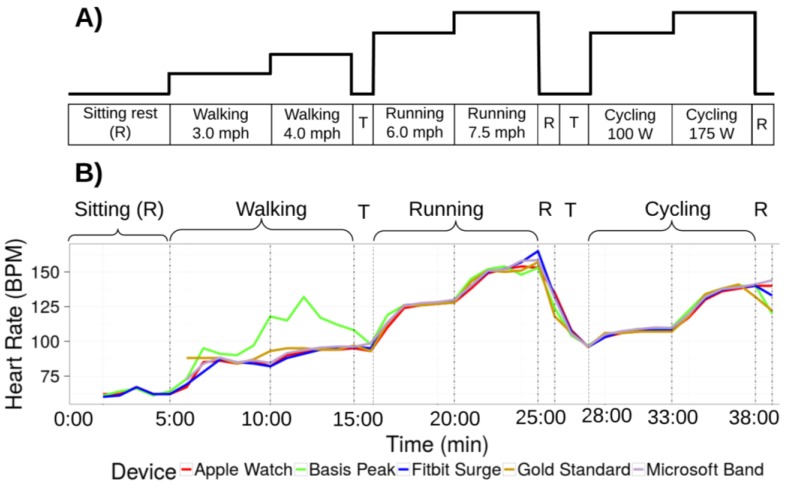
Study protocol. (**A**) Schematic view of the protocol. Participants transition through two intensities of three modalities of exercise as shown. Walking is on a treadmill. Cycling is on a stationary bike. Activities are interspersed with brief (1 min) periods of rest “R”, and transitions between activities are indicated by “T”; (**B**) Data from one participant wearing four devices. Data for the error analysis is derived from the last minute of each stage. Overall, error is within an acceptable range with the exception of the walking phase for one device (green line).

Participants performed the standardized exercise protocol shown in Figure 1 in a controlled laboratory setting. Participants were wearing up to four devices simultaneously and underwent continuous 12-lead electrocardiographic (ECG) monitoring and continuous clinical grade indirect calorimetry (expired gas analysis) using FDA approved equipment (Quark CPET, COSMED, Rome, Italy). After being fitted with all equipment, the protocol started with the participant seated for 5 min. This led to a transition to a treadmill and walking (3.0 mph at 0.5% incline) for 10:00 min followed by faster walking (4.0 mph at 0.5% incline) until 15:00 min, slow running (average speed 5.7 mph at 0.5% incline, range 4.5–6.5 mph) until 20:00 min, and faster running (average speed 6.9 mph at 0.5% incline, range 4.8–9.0 mph) until 25:00. Thereafter, there was 1 min of sitting recovery, and 2 min of rest and transition to a cycle ergometer where 5 min of low intensity cycling (average work rate 88 W, range 50–100 W) until 33:00 min was followed by more intense cycling (average work rate 160 W, range 80–225 W) until 38:00 min, and 1 min of sitting recovery concluded the protocol. Both the running and cycling stages were individualized to the participants’ individual fitness levels in order to maximize range of HR and EE. The last minute of each stage was used for the analysis.

### 2.3. Device Data Collection

Data was collected according to manufacturers’ instructions or by making use of an Application Programming Interface (API).

#### 2.3.1. Apple Watch

All data from the Apple Watch was sent to the Apple Health app on the iPhone, and exported from Apple Health in XML format for analysis. The Apple Health app provided heart rate, energy expenditure, and step count data sampled at one minute granularity. For intense activity (running and max test), the sampling frequency was higher than once per minute. In cases where more than one measurement was collected each minute, the average measurement for the minute was utilized, since the minute average is the granularity for several of the other devices.

#### 2.3.2. Basis Peak (Version 1)

Minute-granularity data was downloaded directly from the Basis app.

#### 2.3.3. Fitbit Surge

The Fitbit Developer API was used to create an application for downloading data at minute-level granularity from the Fitbit Surge device [14] (project key: M2ipOlQ6KOH6nAO4UMjKYmU0AEaSipy0i).

#### 2.3.4. Microsoft Band (Version 1)

The mitmproxy software tool [15] was utilized to extract data from the Microsoft Band, following the technique outlined by J. Huang [16]. Data packets transmitted by the Microsoft phone app were re-routed to an external server for aggregation and analysis. Sampling granularity varied by activity and subject. In cases where multiple data samples were collected each minute, the last data sample for the minute was utilized in the analysis.

#### 2.3.5. Mio Alpha 2

The raw data from the Mio device is not accessible. However, static images of the heart rate over the duration of the activity are stored in the Mio phone app. The WebPlotDigitizer tool was utilized to trace over the heart rate images and to discretize the data to the minute level.

#### 2.3.6. PulseOn

The PulseOn Android application transmits raw data to a SQLite3 database on the Android device. The SQLite3 database stores data sampled at three second granularity. Three-second samples for the last minute of each activity state were averaged to generate heart rate and energy expenditure values for the activity state.

#### 2.3.7. Samsung Gear S2

Raw data from the Samsung Gear is not accessible to users. However, heart rate and step count over time are displayed as static images within the Samsung Gear App. The WebPlotDigitizer [17] tool was utilized to trace over the static images and to discretize them to the minute level.

### 2.4. Statistical Analysis

Statistical analysis was performed separately for HR and EE. The gas analysis data from indirect calorimetry (VO_2_ and VCO_2_) served as the gold standard measurement for calculations of EE (kcal/min). ECG data was used as the gold standard for HR (beats-per-minute; bpm). The percent error relative to the gold standard was calculated for HR and EE using the following formula:

Error = (device measurement−gold standard)/gold standard

Two-way ANOVA with post-hoc Tukey honest significant difference (HSD) was performed to check for a difference between groups for categorical demographic covariates: sex (male/female), arm choice (right/left), device position along the wrist (anterior/posterior) and device error measurements in heart rate (Table S2) and energy expenditure (Table S3). For the continuous demographic variables (age, BMI, Fitzpatrick skin tone, Von Luschan skin tone, VO_2_max, and wrist circumference), a Pearson correlation test was performed between the demographic variable and device error (Tables S4 and S5). This was done with the R “stats” package (version 3.2.2), using the “cor.test” function [18]. A separate test was performed for each device, and *p*-values were adjusted with the Bonferroni correction for multiple testing.

Principal component analysis was performed to identify outliers and to cluster devices by error profiles. Any subjects with missing data were excluded from the principal component analysis (PCA). A singular value decomposition (SVD) was computed over the activity error rates. Variables were not centered, so as to find components of deviation about zero, and the loadings for each principal component were computed.

Several regression approaches were applied to uncover associations in the dataset. The “lm” function from the “statistics” package in R was used to fit a linear regression model [18]. The first principal component from the PCA analysis was the response variable; predictor variables included device, sex, age, BMI, Von Luschen skin tone, and VO_2_max. The correlated variables (height and weight correlated with BMI) and the Fitzpatrick skin tone measure (correlated with the Von Luschen skin tone measure) were excluded from the analysis.

In a parallel approach, a general estimating equation [19] was used to perform a regression analysis with device error as a response variable and device name, activity type, activity intensity, sex, age, height, weight, BMI, skin tone, wrist circumference, and VO_2_max as predictor variables. Interaction terms between the predictor variables of sex and age, activity and device, and intensity and device were included in the analysis. The exchangeable correlation structure was applied to enable inclusion of potentially correlated predictor variables. Regression was performed with the “gee” package in R. The device contrasts were computed relative to the Apple Watch, and the activity contrasts were computed relative to the sitting activity. The “pdredge” function from the “MuMIn” package (version 1.15.6) in R [20] was used to select the optimal subset of predictor variables to regress on the error response.

In a third regression technique, the root mean square error (from zero) was computed for each individual on each device. Regression was then performed with device type as the predictor variable, and the root mean square error values across subjects as the response variable. The Apple Watch served as the base factor value. The effects for other devices served as contrasts with Apple. The “glm” function from the R statistics package was used to fit a gamma distribution.

Finally, a Bland–Altman analysis was performed using the “BlandAltmanLeh” R package [21]. Measurement error relative to gold standard was averaged across all devices for a subject. These averages were plotted relative to the difference in measurement for the given subject/activity across devices (Figure S2).

### 2.5. Error

We determined an error rate of 5% at a *p*-value of 0.05 to be within acceptable limits since this approximates a widely accepted standard for statistical significance, and there is precedent within health sciences research for this level of accuracy in pedometer step counting [22]. To gain a sense of the overall performance of each device for each parameter, a mixed effects linear regression model was utilized, allowing for repeated measures on subjects. This was estimated using the general estimating equation (GEE) approach [19]. The GEE approach was selected due to the ability of this method to account for unknown correlations between the model outcomes. For example, it was unknown a priori whether there was a correlation between device error and any of the subject metadata parameters, such as BMI, sex, or skin tone. The GEE allows the fitting of a linear model to correlated data. First, the device type, activity type, activity intensity, and metadata confounding factors were used as inputs to a general estimating equation, with the magnitude of the error as the output variable. Second, a singular value decomposition of the dataset was performed, treating activity type/intensity as the features. Input variables were not centered, so as to find components of deviation about zero. The contribution of each feature to the first four principal components was computed to determine the degree to which it explained the variation in device measurements.

## 3. Results

### 3.1. Heart Rate (HR)

**Figure 2:**
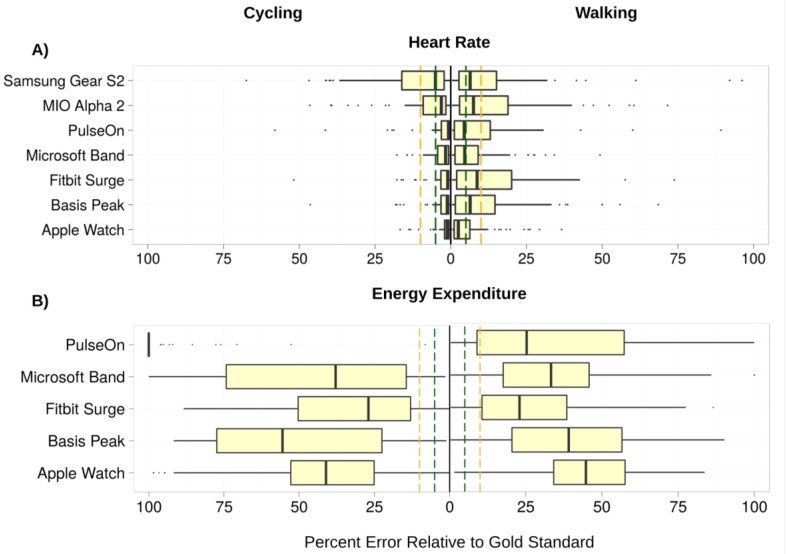
Aggregate relative error (RE) in heart rate (HR) and energy expenditure (EE) for the cycling and walking tasks—the two tasks in the protocol with overall lowest and highest median device error, respectively. Error is calculated as *abs(Gold Standard−Device)/(Gold Standard).* The lower boundary of the boxplots indicates the 25% quantile of data, the middle notch indicates the median data value, and the upper boundary indicates the 75% quantile. Whiskers include all data points that fall within 1.5 interquartile range (IQR) of the 25% and 75% quantile values. Data points that lie further than 1.5 IQR from the upper and lower hinge values are treated as outliers, indicated by black circles. Vertical dashed green lines indicate the 5% error threshold, while the vertical dashed yellow lines indicate the 10% error threshold. Median HR error is below the 5% threshold for all but one device for the cycling task, and below the 10% threshold for all devices on the walking task. EE error rates significantly exceed the 10% threshold for all devices on both the cycling and walking tasks.

**Figure 3:**
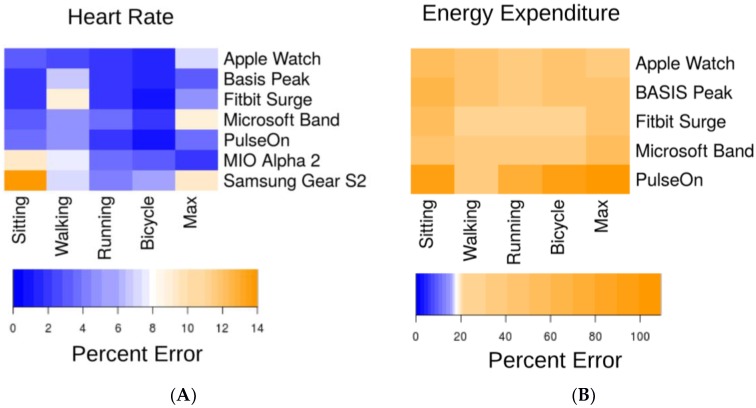
Median device error across activities. We defined an acceptable error range as <5% (dark blue). Light blue, white and yellow shading indicates error outside of this range. (**A**) Median HR beats-per-minute (bpm) error as a percent of the gold standard measurement; (**B**) Median EE (kcal) error as a percent of the gold standard measurement. Note the scaling of the legend color is identical in both panels. Overall, heart rate error was within the acceptable error range for the majority of task/device combinations, but EE error exceeded the allowed threshold for all tasks and devices.

**Figure 4:**
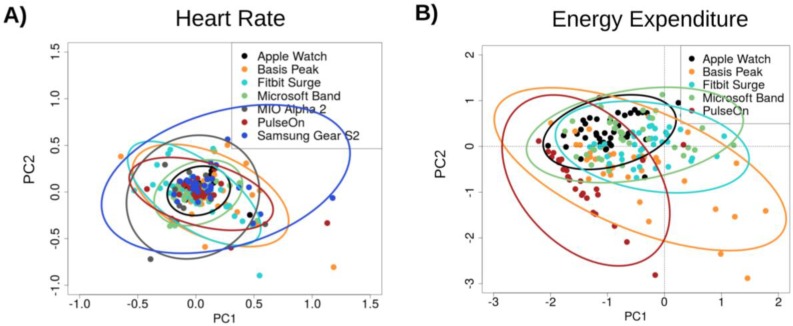
Principal component analysis of device error in (**A**) HR and (**B**) EE. Device errors across all activities (sitting, walking, running, cycling) were aggregated across subjects, excluding any subjects with missing data. The projection of the scaled error values on principal components 1 and 2 (PC2, PC2) are illustrated in the scatter plot, color-coded by device. Ellipses indicate the extent of the first and second principal components that encompass 95 percent of the subject error values for each device. Smaller ellipse area indicates lower variance among device error values, and data points near 0 along the PC1 and PC2 axes indicate low error. The Apple Watch had the most favorable overall error profile while the PulseOn had the least favorable overall error profile.

The lowest error in measuring HR was observed for the cycle ergometer task, 1.8% (0.9%–2.7%) (all results presented as median and 95% confidence interval (CI); Figure 2A), while the highest error was observed for the walking task, 5.5% (3.9%–7.1%). Six of the devices achieved a median error below 5% for HR on the cycle ergometer task; the Samsung Gear S2 achieved a median error rate of 5.1% (2.3%–7.9%). For the walking task, three of the devices achieved a median error rate below 5%: the Apple Watch, 2.5% (1.1%–3.9%); the PulseOn, 4.9% (1.4%–8.6%); and the Microsoft Band, 5.6% (4.9%–6.3%). The remaining four devices had median error between 6.5% and 8.8%. Across devices and modes of activities, the Apple Watch achieved the lowest error in HR, 2.0% (1.2%–2.8%), while the Samsung Gear S2 had the highest HR error, 6.8% (4.6%–9.0%) (Figure 3A and Figure 4A).

### 3.2. Energy Expenditure (EE)

Error in estimation of EE was considerably higher than for HR for all devices (Figure 2B and Figure 3B). Median error rates across tasks varied from 27.4% (24.0%–30.8%) for the Fitbit Surge to 92.6% (87.5%–97.7%) for the PulseOn. For EE, the lowest relative error (RE) rates across devices were achieved for the walking (31.8% (28.6%–35.0%)), and running (31.0% (28.0%–34.0%)) tasks, and the highest on the sitting tasks (52.4% (48.9%–57.0%)).

### 3.3. Error

No evidence was found for a systematic effect of increased error for individuals across tasks or devices. Both principal component analysis and regression via the general estimating equation revealed that activity intensity and sex were significant predictors of error in the measurement of HR. The error rate for males was significantly higher than that for females (*p*-value = 4.56 × 10^−5^, effect size = 0.044, *z* = 3.505 from general estimating equation) across all devices. A Tukey HSD difference of means of 43.5% (ANOVA *p*-value < 1× 10^−5^) was found for the Basis watch; a difference of means of 14.7% (ANOVA *p*-value = 0.0165) was found for the Fitbit. Figure S1 indicates that males had, on average, a 4% higher error in HR across devices and tasks. Higher VO_2_max was significantly associated with HR error on the walking task for the Microsoft Band (*t* = 2.25, *p*-value = 0.03, effect size = 0.004 from the lmfit regression analysis) and the Basis (*t* = 2.34, *p*-value = 0.025, effect size = 0.01 from the lmfit regression analysis). Weight (*t* = −2.53, *p*-value = 0.016, effect size = −0.002), BMI (*t* = −2.78, *p*-value = 0.009, effect size = −0.01), and wrist size (*t* = −2.71, *p*-value = 0.01, effect size = −0.026) were all negatively associated with HR error in the running task for the Apple Watch.

### 3.4. Predictor Variable Associations with Heart Rate and Energy Expenditure Estimation Errors

Variable loadings for the first and second principal component were computed to identify which features contribute most to the variance captured in the PCA analysis results from Figure 4. Variable loadings indicate the degree to which each data feature (activity type or activity intensity) contributes to each principal component. The variable loadings for the first principal component were higher for the less intense forms of activity (sit = 0.37, walk1 = 0.40, walk2 = 0.73, run1 = 0.40, run2 = −0.02, bike1 = −0.02, bike2 = −0.02; Table S6). The gee analysis yielded *p*-value = 1.232 × 10^−6^, effect = −0.027 for the intensity.

The general estimating equation regression identified seven predictor terms that were significantly associated with heart rate device error. In order of significance and using the sitting task and Apple Watch device as the defaults for determining contrasts, they were: Sex (male) (*p*-value = 4.56 × 10^−4^, effect size = 0.074), device: Samsung Gear S2 (*p*-value = 1.385 × 10^−3^, effect size = 0.173), activity: biking (*p*-value = 2.96 × 10^−3^, effect size = −0.041), activity: walk: device: Fitbit Surge (*p*-value = 5.00 × 10^–3^, effect size = 0.079), activity:max test: device: Samsung Gear S2 (*p*-value = 8.35 × 10^−3^, effect size = −0.203), activity: max: device: Mio (*p*-value = 8.97 × 10^–3^, effect size = −0.138), device: Mio (*p*-value = 1.971 × 10^−2^, effect size = 0.062).

## 4. Discussion

There are three principal findings from the current study. In a diverse group of individuals: (1) most wrist-worn monitoring devices report HR with acceptable error under controlled laboratory conditions of walking, running and cycling; (2) no wrist-worn monitoring devices report EE within an acceptable error range under these conditions; (3) of the devices tested, the Apple Watch had the most favorable error profile while the Samsung Gear S2 had the least favorable error profile (Figure S3). This study adds to the literature on wearables by including a sample of highly diverse participants, including skin tone, using FDA approved devices as a gold standard, by developing error models and by proposing a standard for clinically acceptable error.

Our finding that HR measurements are within an acceptable error range across a range of individuals and activities is important for the consumer health environment and practitioners who might be interested to use such data in a clinical setting.

These findings are in agreement with prior work looking at fewer devices in a smaller number of less diverse individuals [23]. In that study, HR error was within 1%–9% of reference standards. In our study, six of the seven devices evaluated had a median HR error for the most stable activity, cycling, of below 5%. Covariates such as darker skin tone, larger wrist circumference, and higher BMI were found to correlate positively with increased HR error rates across multiple devices. Device error was lower for running vs. walking but higher at higher levels of intensity within each modality.

In contrast with low reported error for HR measurement, no device met our prespecified error criterion for energy expenditure. This finding is also in agreement with a previous smaller study [23] where EE estimates were up to 43% different from the reference standard. It is not immediately clear why EE estimations perform so poorly. While calculations are proprietary, traditional equations to estimate EE incorporate height, weight, and exercise modality. It is likely that some algorithms now include HR. Since height and weight are relatively fixed and HR is now accurately estimated, variability likely derives either from not incorporating heart rate in the predictive equation or from inter-individual variability in activity specific EE. There is evidence for this—for example, 10,000 steps have been observed to represent between 400 kilocalories and 800 kilocalories depending on a person’s height and weight [24].

Since devices are continually being upgraded and algorithms tuned, we created a website for sharing validation data for the community and to provide a forum for users to interact with the most up-to-date performance evaluations from this ongoing study (http://precision.stanford.edu/) [23,25,26,27,28]. While the FDA currently considers consumer wearable sensors such as wrist-worn devices as low risk (Class 1) and therefore not subject to active regulation, [29] they are however expected to increasingly inform clinical decision making. This makes transparency regarding benefits and limitations of paramount importance.

### Limitations

Our study has limitations. We only tested devices and algorithms that were available at the time of our study. Laboratory validation of wearable devices is a logical first step toward determining whether commercial wearables have potential use for medical applications. However, the true potential of such wearables lies in their ability to provide continuous real-time monitoring outside of the clinic. This will be the focus of future research.

## 5. Conclusions

We assessed, in a controlled laboratory setting, the reliability of seven wrist-worn devices in a diverse group of individuals performing walking, running and cycling at low and high intensity. We found that in most settings, heart rate measurements were within acceptable error range (5%). In contrast, none of the devices provided estimates of energy expenditure that were within an acceptable range in any setting. Individuals and practitioners should be aware of the strengths and limitations of consumer devices that measure heart rate and estimate energy expenditure. We encourage transparency from device companies and consistent release of validation data to facilitate the integration of such data into clinical care. We provide a forum for the community to share such data freely to help achieve this end.

## Figures and Tables

**Table 1:** Participant characteristics. Values are means (min–max), standard deviation (sd). Skin tone rating by Fitzpatrick scale. VO_2_max (maximal oxygen uptake) was either measured at incremental test to exhaustion or estimated from submaximal cycling using the Åstrand nomogram.

	Men (*n* = 29)	Women (*n* = 31)
Age (years)	40 (21–64, sd = 11.48)	37 (23–57, sd = 9.77)
Body mass (kg)	80.1 (53.9–130.6, sd = 13.25)	61.7 (47.8–89.2, sd = 12.91)
Height (cm)	179.0 (159.1–190.0, sd = 7.81)	165.9 (154.4–184.2, sd = 7.90)
Body mass index (kg/m^2^)	24.9 (20.7–39.3, sd = 3.46)	22.4 (17.2–28.8, sd = 3.31)
Skin tone (scale 1–6)	3.7 (1–5, sd = 1.39)	3.7 (1–6, sd = 1.25)
Wrist circumference (cm)	17.3 (16.0–21.0, sd = 1.11)	15.4 (13.5–17.5, sd = 1.30)
VO_2_max (ml/kg/min)	52.8 (38.2–66.6, sd = 8.48)	45.3 (31.7–56.5, sd = 7.62)

## Supplementary Materials

The following are available online at www.mdpi.com/2075-4426/7/2/3/S1, Figure S1: All device error measurements collected in the study, grouped by subject, Figure S2: Bland-Altman plot of error across activities, Figure S3: Diversity of skin tone among study participants as measured by the Von Luschan chromatic scale, Table S1: Firmware and software versions of fitness trackers and associated phone applications, Table S2: Tukey post-hoc test results for categorical demographic variables as predictors of device error in heart rate measurement, Table S3: Tukey post-hoc test results for categorical demographic variables as predictors of device error in energy expenditure measurement, Table S4: 2-Tailed pearson correlation test of heart rate percent error with covariates, Table S5: 2-Tailed pearson correlation test of energy expenditure percent error with covariates, Table S6: Feature loadings for the first four principal components in the heart rate PCA.
