# Supplementary material for: Accuracy in Wrist-Worn, Sensor-Based Measurements of Heart Rate and Energy Expenditure in a Diverse Cohort
Source: J Pers Med. 2017 May 24;7(2):3. doi: 10.3390/jpm7020003 (PMC5491979; doi:10.3390/jpm7020003)
Supplement: Supplementary file 1 [file jpm-07-00003-s001.pdf]

Supplementary Materials

Device	Firmware Version	Software Version
**Microsoft Band (v. 1)**	10.3.3304.0 09 R	1.3.10901.1
**Basis Peak (v.2)**	build 14.6.0	1.15.0
**Fitbit Surge**	16.31.6.3	2.10.1
**Apple Watch**	1.0.1	123632
**Mio**	01.01	2.4.5
**PulseOn**	2.36	1.1.9541JP
**Samsung Gear**		R720XXU2AOIL

Supplementary Table 1: Firmware and software versions of fitness trackers and associated phone applications.

Heart Rate Percent Error vs. Covariate ANOVA

	Male-Female		Right – Left Arm		Proximal (Near Wrist) – Distal (higher on arm)	
**Watch**	**ANOVA *p*-value**	**difference means (Tukey HSD)**	**ANOVA *p*-value**	**difference means (Tukey HSD)**	**ANOVA *p*-value**	**difference means (Tukey HSD)**
**Apple**	**0.587**	**−0.0039**	**0.0138**	**0.0843**	**0.0384**	**0.0164**
**Basis**	**0.713**	**0.0066**	**0.28**	**0.0199**	**0.109**	**0.0291**
**Microsoft**	**0.612**	**0.013**	**0.348**	**−0.0165**	**0.0655**	**−0.0325**
**Fitbit**	**0.354**	**−0.015**	**0.767**	**−0.00548**	**0.216**	**0.0234**
**Samsung**	**0.755**	**0.0102**	**0.0877**	**−0.0581**	**0.0954**	**0.0561**
**Mio**	**0.489**	**0.0186**	**0.142**	**0.0403**	**0.826**	**0.00609**
**Pulseon**	**0.787**	**−0.0076**	**0.657**	**−0.0133**	**0.0103**	**−0.074**

Supplementary Table 2. Tukey post-hoc test results for categorical demographic variables as predictors of device error in heart rate measurement. Sex (male/female), arm choice (right/left), wrist position (proximal/distal) are categorical input variables, while device error values are the continuous dependent variables. A separate ANOVA analysis and Tukey HSD test was performed for each device, and the ANOVA *p*-value column has been corrected (Bonferroni) for the number of devices that were tested.

Energy Percent Error vs. Covariate ANOVA

	Male-Female		Right – Left Arm		Proximal (Near Wrist) – Distal (higher on arm)	
**Watch**	**ANOVA *p*-value**	**difference means (Tukey HSD)**	**ANOVA *p*-value**	**difference means (Tukey HSD)**	**ANOVA *p*-value**	**difference means (Tukey HSD)**
**Apple**	**0.152**	**0.0729**	**0.389**	**0.0426**	**0.389**	**0.0426**
**Basis**	**0**	**0.435**	**0.0658**	**−0.109**	**0.0257**	**0.13**
**Microsoft**	**0.0874**	**0.107**	**0.241**	**−0.0738**	**0.0489**	**0.123**
**Fitbit**	**0.0165**	**0.147**	**0.757**	**−0.018**	**0.00891**	**−0.155**
**Pulseon**	**0.135**	**0.195**	**0.671**	**−0.0591**	**0.919**	**0.0136**

Supplementary Table 3. Tukey post-hoc test results for categorical demographic variables as predictors of device error in energy expenditure measurement. Sex (male/female), arm choice (right/left), wrist position (proximal/distal) are categorical input variables, while device error values are the continuous dependent variables. A separate ANOVA analysis and Tukey HSD test was performed for each device, and the ANOVA *p*-value column has been corrected (Bonferroni) for the number of devices that were tested.

	Age			BMI			Fitzpatrick skin tone			Von Luschan skin tone			VO_2max			Wrist circumference		
**Watch**	*z*-score	*p*-value	Pearson r	*z*-score	*p*-value	Pearson r	*z*-score	*p*-value	Pearson r	*z*-score	*p*-value	Pearson r	*z*-score	*p*-value	Pearson r	*z*-score	*p*-value	Pearson r
**Apple**	0.796	0.427	0.061	0.581	0.562	0.045	−0.471	0.639	−0.036	−0.386	0.7	−0.03	−0.946	0.346	-0.073	0.04	0.968	0.003
**Samsung**	1.874	0.063	0.147	0.739	0.461	0.059	2.011	0.046	0.158	2.293	0.023	0.179	−1.773	0.078	-0.14	-0.653	0.515	−0.052
**Pulseon**	−3.854	0	−0.319	−2.856	0.005	-0.242	−0.888	0.376	−0.077	−1.321	0.189	−0.115	1.322	0.188	0.115	−1.883	0.062	−0.162
**Fitbit**	−0.502	0.616	−0.039	−0.278	0.781	-0.021	2.027	0.044	0.155	2.232	0.027	0.17	−2.644	0.009	−0.2	−1.847	0.066	−0.141
**Basis**	−1.695	0.092	−0.13	0.516	0.606	0.04	3.498	0.001	0.261	3.782	0	0.28	−2.683	0.008	−0.203	−0.826	0.41	−0.064
**Mio**	−0.118	0.906	−0.011	−1.138	0.257	-0.103	1.056	0.293	0.096	1.145	0.255	0.104	1.918	0.057	0.172	−0.451	0.653	−0.041
**Microsoft**	−1.013	0.312	−0.078	−0.296	0.767	-0.023	0.69	0.491	0.053	0.617	0.538	0.047	−0.106	0.915	−0.008	-0.016	0.987	−0.001
**Device Mean**	−1.288	0.198	−0.07	−0.332	0.74	-0.018	1.369	0.172	0.074	1.441	0.15	0.078	−1.332	0.184	−0.072	−1.105	0.27	−0.06

Supplementary Table 4: 2-Tailed Pearson correlation test of heart rate percent error with covariates. All *p*-values reported are Bonferroni-corrected for the number of tests that were performed.

	Age			BMI			Fitzpatrick skin tone			Von Luschan skin tone			v02max			Wrist circumference		
**Watch**	***z*-score**	***p*-value**	**Pearson r**	***z*-score**	***p*-value**	**Pearson r**	***z*-score**	***p*-value**	**Pearson r**	***z*-score**	***p*-value**	**Pearson r**	***z*-score**	***p*-value**	**Pearson r**	***z*-score**	***p*-value**	**Pearson r**
**Apple**	0.449	0.654	0.034	0.842	0.401	0.064	0.34	−0.011	0.991	−0.001	0.734	0.026	2.911	0.004	0.217	2.24	0.026	0.168
**PulseOn**	−0.152	0.879	-0.013	2.653	0.009	0.227	0.389	0.27	0.788	0.024	0.698	0.034	−0.679	0.498	-0.06	2.887	0.005	0.246
**Fitbit**	0.247	0.805	0.019	2.572	0.011	0.194	0.288	0.787	0.432	0.06	0.774	0.022	1.529	0.128	0.116	2.199	0.029	0.166
**Basis**	1.032	0.304	0.078	4.459	0	0.322	0.882	1.255	0.211	0.095	0.379	0.067	5.412	0	0.381	7.179	0	0.48
**Microsoft**	0.803	0.423	0.062	1.413	0.16	0.108	−1.058	−1.178	0.24	-0.09	0.291	-0.081	3.723	0	0.275	2.338	0.021	0.177
**Device Mean**	0.425	0.671	0.024	3.705	0	0.208	0.388	0.456	0.649	0.026	0.698	0.022	1.318	0.188	0.075	4.249	0	0.236

Supplementary Table 5: 2-Tailed Pearson correlation test of energy expenditure percent error with covariates**.** All *p*-values reported are Bonferroni-corrected for the number of tests that were performed.

	Heart Rate				Energy Expenditure			
**Component**	PC1	PC2	PC3	PC4	PC1	PC2	PC3	PC4
**sit**	-0.37	0.74	0.37	0.39	0.43	0.25	0.57	-0.54
**walk1**	-0.4	0.4	-0.58	-0.49	0.36	-0.66	-0.45	-0.47
**walk2**	-0.73	-0.5	0.36	-0.05	0.24	-0.22	0.08	0.3
**run1**	-0.4	-0.18	-0.46	0.28	0.38	-0.37	0.44	0.35
**run2**	0.02	-0.04	-0.25	0.62	0.12	-0.23	0.13	0.44
**bike1**	0.02	0	-0.09	0.11	0.54	0.44	-0.51	0.29
**bike2**	0.02	-0.11	-0.34	0.36	0.43	0.27	-0.02	-0.04

Supplementary Table 6: Feature loadings for the first four principal components in the heart rate PCA.

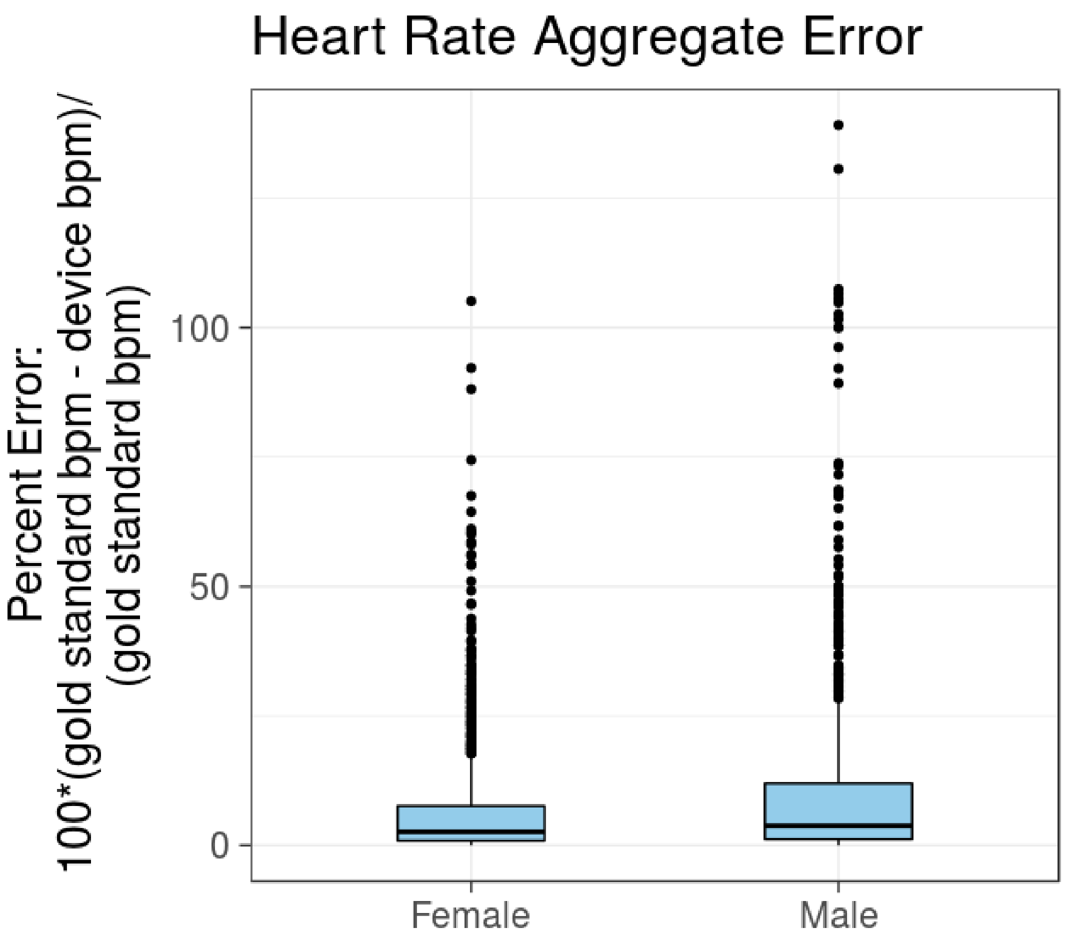

Supplementary Figure 1: All device heart rate error measurements collected in the study, grouped by subject sex. The boxplots indicate the 25% quantile, median, and 75% quantiles of the error measurements. Data points more than 1.5 IQR values above the 75% quantile are treated as outliers and indicated with a dot. Percent error refers to (gold standard bpm – device bpm)/gold standard bpm.

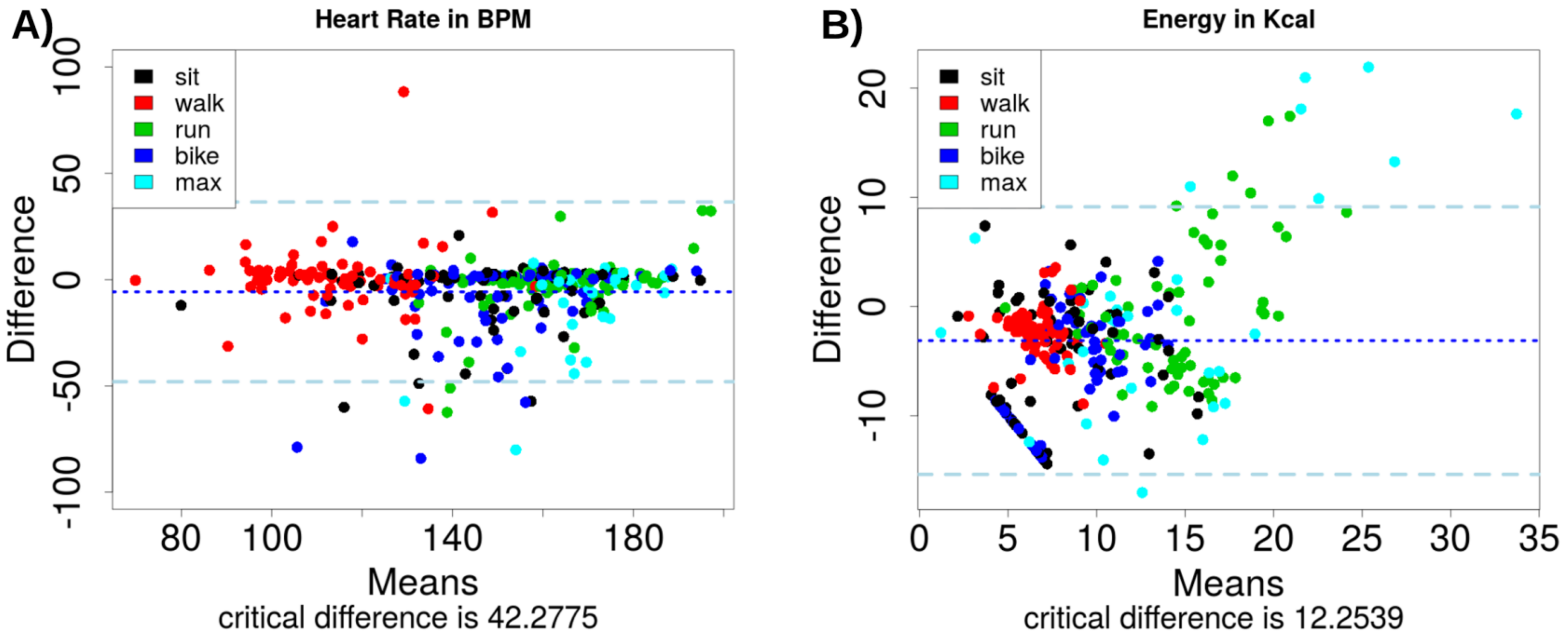

Supplementary Figure 2: Bland–Altman plot of error across activities. a) Heart-rate error in beats per minute, averaged across devices, compared to the gold standard (12-lead ECG). b) Energy expenditure error in Kcal, averaged across devices, compared to the gold standard (gas analysis from indirect calorimetry).

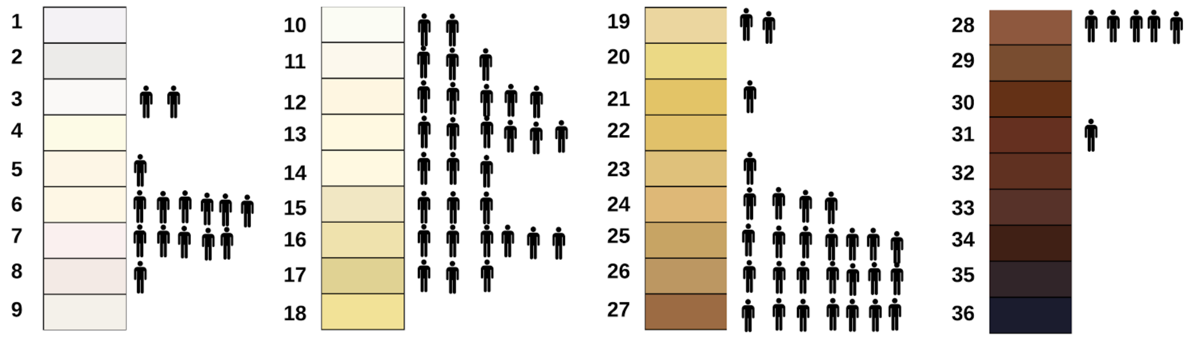

Supplementary Figure 3: Diversity of skin tone among study participants as measured by the Von Luschan chromatic scale. Skin tone shades were labeled 1–36 and the closest matching tone was recorded for each individual.
